# Skin disorders among children living in orphanage centres in Dar es Salaam, Tanzania

**DOI:** 10.1186/s41182-020-00216-9

**Published:** 2020-04-30

**Authors:** Mwanaidi Amiri, Francis F. Furia, Muhammad Bakari

**Affiliations:** 1 Department of Paediatrics and Child Health, School of Medicine, Muhimbili University of Health and Allied Sciences, Dar es Salaam, Tanzania; 2 Department of Internal Medicine, School of Medicine, Muhimbili University of Health and Allied Sciences, Dar es Salaam, Tanzania

**Keywords:** Skin disorders in children, Orphanage centres, Skin disorder in Tanzania

## Abstract

**Background:**

Skin conditions contribute significantly to the global burden of diseases and are among the leading causes of non-fatal disease burden. Children living in orphanage centres are vulnerable to several conditions including dermatological disorders, and there is limited data on the burden of these conditions among orphans in Tanzania. This study was carried out to determine the pattern of dermatological conditions and contributing factors among orphans in Dar es Salaam, Tanzania.

**Methodology:**

A cross-sectional study was conducted among 420 children aged less than 18 years from 12 orphanage centres in Dar es Salaam. Guided interviews using structured questionnaires were carried out to obtain socio-demographic and clinical data from participants. Clinical examination was performed for each participant and whenever indicated skin scrapings and biopsy were obtained.

**Results:**

Four hundred and twenty participants were recruited out of which 281 (66.9%) were male, mean and median ages of participants were 11 ± 3.7 and 12 years, respectively. Two hundred and twenty-five (53.6%) participants were aged between 6 and12 years. Proportion of children with dermatological manifestations among participants was 57.4%. Two hundred and ninety-six diagnoses were made comprising of 192 (64.9%) infections and 104 (35.1%) non-infectious conditions. Tinea capitis was the commonest infection while acne vulgaris was the most common non-infectious condition. Proportionately more male children were affected as compared to female ones, *p* = 0.006.

**Conclusion:**

Skin conditions are common among children living in orphanage centres in Dar es Salaam. Infectious conditions were predominant conditions and male children were more affected than female children. Reducing crowding and improving hygienic practices in these centres will be important in reducing the burden of these conditions.

## Background

Skin conditions contribute significantly to the global burden of non-fatal diseases [1, 2]. The burden of skin disorders is significantly borne by children, and many visits to primary health facilities are attributed to these conditions [3]. Common childhood skin disorders reported in sub-Saharan African (SSA) include pyoderma, tinea capitis, scabies, viral skin infection (mainly molluscum contagiosum), pediculosis pedis and dermatitis [3].

Infections are the commonest skin disorders affecting children in sub-Saharan Africa and include fungal, bacterial and viral infections. Fungal and parasitic infections accounted for 25.4% and 21.4%, respectively for skin conditions reported by Bisek et al. among children in rural setting in Cameroun [4]. Similar observation was reported in Ivory Coast and Nigeria by Yotsu and Ayanlowo, respectively, in studies conducted among children [5, 6].

Skin disorders are common among children in Tanzania as was noted by Komba et al. in a study conducted among children in Dar es Salaam reporting a prevalence of 57.3%. In another study conducted in Northern Tanzania, Kipromo et al. reported 407 skin conditions being diagnosed in 340 children in Northern Tanzania [7, 8]. There is limited information on the burden of skin disorders among orphans in SSA region; orphans are vulnerable to many psychosocial and medical problems including skin disorders [9–11]. The aim of this study was to determine the burden, pattern and factors associated with skin disorders among children living in orphanage centres.

## Methods

### Study design

This was a cross-sectional study conducted among children living in orphanage centres in Dar es Salaam, Tanzania, between May and December 2013.

### Study area

This study was conducted in 12 randomly selected orphanage centres out of 30 in Dar es Salaam city. The city had three administrative municipalities: Kinondoni, Ilala and Temeke. Twenty centres were located in Kinondoni, while Ilala and Temeke had 5 each; out of 30 centres, 16 were officially registered. The total number of children in all centres was 1968 (287 in Temeke, 408 in Ilala and 1303 in Kinondoni). Children were permanently staying in the centres and had no interaction with street children. The total number of children in the 12 centres from which participants were recruited was 757.

### Study population

A total of 420 children aged below 18 years living in orphanage centres were recruited in this study. A minimum sample size of 376 children, this was calculated based on the prevalence of 57.3% of dermatological conditions among primary school children in Dar es Salaam which was reported by Komba et al. [7].

### Sampling technique

Twelve orphanage centres were randomly selected out of 30 centres in Dar es Salaam.

A list of 20 orphanage centres from Kinondoni municipality was made and 8 centres were selected, and two centres each were selected from Temeke and Ilala out of 5 centres in each of the two municipalities. Out of all 420 children, 58 were selected from Temeke centres, 88 from Ilala and 274 from Kinondoni centres. Random sampling was carried out to select participants for this study; data collection including examination of participants was performed after selection. An average of 20 children were recruited per day and all children were given unique identification number to avoid double recruitment. The recruitment was carried out 2 days a week that were Saturdays and Sundays only.

### Data collection

One of the investigators (MA) who was a senior paediatric resident and two research assistants (medical doctors) were trained for 8 weeks in the paediatric dermatology clinic at Muhimbili National Hospital (MNH) by dermatologists before starting data collection. Paediatric dermatology clinics were conducted every Thursday and the average number of patients on each clinic was 15 to 20.

Recruitment of study participants was carried out on Saturdays and Sundays from 9.30 a.m. to 3.00 p.m. for 3 consecutive months. Investigator and research assistants carried out recruitment; dermatological examination was carried out by investigator (MA) or by research assistants under supervision of the investigator. Complete dermatological examination was performed under adequate natural light using a magnifying lens. Diagnosis of most conditions was made clinically with laboratory testing performed for few participants. Five participants were taken to a dermatologist for examination and diagnosis and 30 (7%) of participants were photographed and their diagnoses were made after review of images by a dermatologist at MNH.

A structured questionnaire was used to collect data, which included age, sex and hygiene practice; bathing frequency; use of soap; washing and ironing of clothing; and sharing of bed. Findings of dermatological examination and nutrition assessment were also recorded. Results of tissue processed for histological diagnosis were recorded into the questionnaire.

### Laboratory investigations

#### Specimen collection

Tissue specimens were collected from participants with skin lesions that required laboratory examination; these specimens included skin scraping and biopsy. Scrapings were collected using blunt side of a surgical blade while biopsy was obtained using a 3.5 mm punch. Skin scrapings were taken from one out of four participants who had scabies, this participant had atypical lesion because of superimposed bacterial infection. Skin biopsies were taken from two participants who were suspected to have psoriasis and impetiginized eczema, respectively. Tissue specimens were processed in the Central Pathology Laboratory (CPL) at MNH.

#### Nutritional status assessment

All participants were weighed using a standard beam balance (SECA®). Participants were instructed to put on light clothing and no shoes. For older participants, weight was taken while standing on a weighing scale (SECA). Weight was measured to the nearest 10 g. Calibration of weighing scale to zero was performed on each day of recruitment**.**

Height of participants was measured using either a length board or stadiometre. Caregivers assisted in removing shoes and gently laying the child in supine position on the board, with their heads placed at 90° to the fixed head piece. Research assistants straightened legs of the child at the knees to ensure that feet were at right angle to the sliding foot piece, which was brought into contact with the child’s heels. For those participants who could stand by their own, a height metre was fixed on the wall to measure their height in centimetres.

Nutritional status was assessed through the determination of body mass index (BMI). This was calculated according to the Quetelet equation for all children to ensure uniformity of interpretation. Patient’s body weight in kilogrammes (kg) was divided by his/her height in metres squared (m^2^) [BMI = Weight (kg)/Height (m^2^)]. WHO Body mass index for age *Z* scores for boys and girls, respectively, was used for interpretation.

#### Data entry and analysis

Statistical Package for Social Sciences (SPSS) version 20 was used for data entry, cleaning and analysis. Data were then summarized into frequency distribution two-way tables. Using frequencies, percentages and means/medians where appropriate, descriptive analysis was carried out. Chi-square and Fisher’s exact tests were used to determine the association between categorical variables. Univariate and multivariate analyses were to determine factors associated with dermatologic conditions. *p* value of < 0.05 was considered statistically significant.

### Ethical considerations

Muhimbili University of Health and Allied Sciences (MUHAS) Institutional Review Board approved this study. Permission to conduct this study was sought from respective Municipal Social Welfare Officers. Permission was also sought from the administrators of all orphanage centres who were informed about the purpose of the study before recruiting participants. Written consents were obtained from children’s guardians and assents were sought from children aged 8 years and above. Privacy was observed at the time of dermatological examinations. Children diagnosed to have dermatologic conditions were given appropriate advice and prescriptions were given to orphanage administration for medications.

## Results

### Participants’ socio-demographic and general characteristics

A total of 420 participants were recruited in this study out of which 281 (66.9%) were male; male to female ratio was 2:1. Mean and median ages of participants were 11 ± 3.7 and 12 years, respectively. Two hundred and twenty-five (53.6%) participants were aged between 6 and 12 years and only 31 (7.4%) were not attending school. Two hundred and ninety-three (69.8%) children had normal nutritional status (Table 1).

**Table 1:** Socio-demographic characteristics and nutritional status of children living in orphanage centres in Dar es Salaam

Variables	Number (%)
**Sex**	
Male	281 (66.9)
Female	139 (33.1)
**Age groups (years)**	
0-5	29 (6.9)
6-12	225 (53.6)
> 12	166 (39.5)
**School attendance**	
Yes	389 (92.6)
No	31 (7.4)
**Nutritional status**	
Overweight	45 (10.7)
Normal	293 (69.8)
Underweight	82 (19.5)

### Prevalence of dermatologic conditions

A total of 241 (57.4%) children were diagnosed to have dermatological conditions, and 296 diagnoses were made from which 192 (64.9%) were infectious and 104 (35.1%) were non-infectious conditions. One hundred and eighty-eight children (78.0%) had one dermatologic diagnosis, while 39 (16%) had two dermatologic diagnoses. Twelve children (5%) and 2 children (1%) had three and four dermatologic diagnoses, respectively.

Male participants were noted to have higher prevalence of superficial fungal infections (43.1%) as compared to female participants (27.3%), *p* = 0.002, and children aged 6–12 years had significantly higher prevalence of superficial fungal infections (44.4%) as compared to those aged 0–5 and > 12 years who had prevalence rates of 27.6% and 30.7%, respectively, *p* = 0.011 (Tables 3 and 4).

### Patterns of dermatological conditions

Superficial fungal infections contributed to 94% of all infectious conditions, with tinea capitis accounting for (63.5%) followed by pityriasis versicolor (17.7%). Parasitic, viral and bacterial infections contributed to 5.7% of all infectious conditions as shown in Table 2.

**Table 2:** Pattern of infectious dermatological conditions among children living in orphanage centres in Dar es Salaam (*n* = 192)

Dermatological conditions	Frequency	%
**Parasitic**		
Scabies	4	2.08
**Fungal**		
Tinea capitis	115	59.9
Tinea corporis	26	13.5
Tinea pedis	2	1.04
Pityriasis versicolor	34	17.7
Candidiasis	4	2.08
**Bacterial**		
Scalp folliculitis	1	0.52
Impetigo	2	1.04
Furunculosis	3	1.56
**Viral**		
Herpes simplex labialis	1	0.52

Acne vulgaris was the most common non-infectious condition reported by 23 (22.1%) participants followed by inflammatory hypo and hyperpigmented lesions, hypertrophic scars and atopic eczema which were noted in 19 (18.3%), 15 (14.4%) and 11 (10.6%), respectively (Table 3).

**Table 3:** Pattern of non- infectious dermatological conditions among children living in orphanage centres in Dar es Salaam (*n* = 192)

Dermatological conditions	Frequency	%
**Eczema and related conditions**		
Atopic eczema	11	10.6
Lip licking dermatitis	2	1.9
Keratosis pilaris	3	2.9
Scalp seborrheic dermatitis	5	4.8
Prurigo nodularis	1	0.96
Papular urticaria	10	9.6
Pruritic papular eruption	1	0.96
Irritant contact dermatitis	3	2.9
**Papulosquamous disorders**		
Psoriasis	1	0.96
**Adnexal disorders**		
Acne Vulgaris	23	22.1
Traction Alopecia	1	0.96
**Genodermatoses**		
Neurofibromatosis	1	0.96
**Scars**		
Hypertrophic scars	15	14.4
Keloid	1	0.96
**Others**		
Nevus	7	6.7
Post-inflammatory hyper- and hypopigmentation	19	18.3

### Factors associated with presence of dermatological conditions

Male children were noted to be more affected by dermatologic conditions (62.6%) more than female children (46.5%). All the other factors assessed did not show statistically significant difference (Table 4).

**Table 4:** Factors associated with dermatological conditions among children living in orphanage centres in Dar es Salaam

Characteristics	Categories	Total	Skin lesions, ***n*** (%)	***p*** value
**Sex**	Male	281	176 (62.6)	0.002
	Female	139	65 (46.5)	
**Age groups (years)**	0–5	29	17 (58.6)	0.806
	6–12	225	132 (58.7)	
	> 12	166	92 (55.4)	
**Bathing frequency**	Once	28	16 (57.1)	0.995
	Twice	271	156 (57.6)	
	More than twice	121	69 (57.0)	
**Use of soap**	Yes	419	241 (57.5)	0.245
	No	1	0 (0.0)	
**Ironing clothes**	Yes	96	59 (61 .5)	0.358
	No	361	182 (56.2)	
**Sharing garments**	Yes	76	42 (55.3)	0.680
	No	344	199 (57.8)	
**Use of shoes**	Yes	416	238 (57.2)	0.474
	No	4	3 (75.0)	
**Change of garments**	Daily	314	179 (57.0)	0.144
	Every other day	78	50 (64.1)	
	More than 1 day	28	12 (42.9)	
**Meals per day**	3	314	177 (56.4)	0.471
	> 3	106	64 (60.4)	
**Number per bed**	1	196	113 (57.7)	0.916
	2	224	128 (57.1%)	
**Change of bed sheets**	Daily	18	12 (66.7%)	0.400
	Once per week	396	227 (57.3%)	
	< Once per week	6	2 (33.3%)	

As described in Table 5, the odds of female children to be infected with superficial fungal infection were less by 50% as compared to male children. Similarly, the odds of having superficial fungal infection was 1.8 times more likely in children aged between 6 and 12 years as compared to children aged more than 12 years (odds ratio was 1.8 and 95% CI was between 1.2 and 2.8), *p* = 0.006. Male gender and age between 6 and 12 years were noted to be independent predictors of dermatologic manifestations in this study as described in Table 6.

**Table 5:** Univariate analysis of fungal infections in relation to socio-demographic and hygienic habits of children living in orphanage centres, Dar es Salaam (*n* = 420)

Characteristics	Categories	Total	***N*** (%) SFI; ***n*** = 159	Superficial fungal infections, OR (95%CI)	***p*** value
**Sex**	Female	281	38 (27.3)	0.5 (0.3–0.8)	0.002
	Male	139	121 (43.1)	Referent	
**Age groups (years)**	0–5	29	8 (27.6)	0.9 (0.4–2.1)	0.735
	6–12	225	100 (44.4)	1.8 (1.2–2.8)	0.006
	> 12	166	51 (30.7)	Referent	
**Bathing frequency**	Once	28	11 (39.3)	1.4 (0.6–3.3)	0.425
	Twice	271	110 (40.6)	1.5 (0.9-2.4)	0.084
	> Twice	121	38 (31.4)	Referent	
**Use of soap**	Yes	419	159 (37.9)	_	
	No	1	0 (0.0)	_	
**Iron clothes**	Yes	96	39 (40.6)	1.2 (0.7–1.9)	0.525
	No	361	120 (37.0)	Referent	
**Sharing garments**	Yes	76	30 (39.5)	1.1 (0.7–1.9)	0.748
	No	344	129 (37.5)	Referent	
**Use of shoes**	Yes	416	159 (38.2)	_	
	No	4	0 (0.0)	_	
**Change of garments**	Daily	314	115 (36.6)	1.4 (0.6–3.4)	0.397
	Every other day	78	36 (46.2)	2.1 (0.8–5.4)	0.109
	More than 1 day	28	8 (28.6)	Referent	
**Meals per day**	3	314	119 (37.9)	1.0 (0.6–1.6)	0.976
	> 3	106	40 (37.7)	Referent	
**Number per bed**	1	196	73 (37.2)	1.0 (0.6–1.4)	0.809
	2	224	86 (38.4)	Referent	
**Change of bed sheets**	Daily	18	8 (44.4)	1.2 (0.2–6.7)	0.830
	Once/week	396	149 (37.6)	1.6 (0.2–11.1)	0.634
	< Once/week	6	2 (33.3)	Referent

**Table 6:** Multivariate analysis of commonly encountered dermatological conditions among children living in orphanage centres, Dar es Salaam

Characteristics	Categories	Total	No. (%) with SFI; ***n*** = 159	Superficial fungal infections, adjusted OR (95%CI)	***p*** value
**Sex**	Female	281	38 (27.3)	0.5 (0.3–0.8)	0.004
	Male	139	121 (43.1)	Referent	
**Age groups (years)**	0–5	29	8 (27.6)	1.0 (0.4–2.4)	0.925
	6–12	225	100 (44.4)	1.9 (1.2–3.0)	0.006
	> 12	166	51 (30.7)	Referent	
**Bathing frequency**	Once	28	11 (39.3)	1.0 (0.4–2.4)	0.986
	Twice	271	110 (40.6)	1.0 (0.6–1.7)	0.881
	> Twice	121	38 (31.4)	Referent	
**Change of garments**	Daily	314	115 (36.6)	1.5 (0.6–3.6)	0.406
	Every other day	78	36 (46.2)	1.9 (0.7–4.8)	0.204
	More than 1 day	28	8 (28.6)	Referent	

## Discussion

Skin disorders were noted to be common among children living in orphanage in Dar es Salaam with a prevalence of 57.4%, with males being more affected than females. These findings are similar to findings reported earlier among school children in Tanzania by Komba et al. (57.3%) and Ferie et al. (55%) indicating equal burden of skin disorders among children in general population and orphanage centres [7, 12]. A slightly lower prevalence of 40.9% was reported by Khalifa et al. among children in Iran which might be attributed to environmental and geographical differences and health seeking behaviours between the two settings [13].

Infections were the predominant skin conditions in this study accounting for (64.9%), which is consistent with other reports among children, a study conducted among children who presented to dermatology clinic in Northern Tanzania reported similar findings with predominance of infections and infestations [8]. Studies from India and Pakistan reported similar among children [14–16].

Predominance of infectious conditions noted from studies conducted in Tanzania and other developing countries may signify the level of hygienic status, which is low with poor socio-economic status. This is further supported by higher burden of non-infectious skin conditions reported from studies conducted in countries with higher socio-economic status including Iraq, Switzerland, Turkey and Kuwait [13, 17–19].

In this study superficial fungal infection accounted for 94% of the infectious conditions with predominance of tinea capitis, which is similar to findings from studies reported from Egypt (78.6%), Nigeria (87.9%) and India (65.5%) [14, 20, 21]. Male children were more affected with fungal infections as compared to female children, which might be attributed to differential hygienic practices. Sharing of clothing reported in this study, short hair of children and humid climatic condition in Tanzania are plausible reasons for high proportion of fungal infections noted in this study [22].

An earlier study conducted in Tanzania among primary school children reported children aged 6–12 years to be more affected by tinea capitis as was the case in this study, this is possibly due to increased interaction among younger children [7]. Older children in this study were predominantly affected by pityriasis versicolor consistent with other reports from the region [7, 22, 23]. Older children have increased activities of sebaceous glands as a result of puberty facilitating this type of fungal infection.

Scabies was the only parasitic infestation noted in this study affecting 2.1% of children. This finding is similar to reports from several other studies conducted among children in the region. Scabies was reported in 3.7% and 2.8% among children in Ivory Coast and Cameron, respectively [4, 5]. Low prevalence rates were also reported in Egypt, Kuwait and Switzerland [17, 19, 24].

Post-inflammatory hypo- and hyperpigmentation, acne vulgaris and atopic dermatitis were the predominant non-infectious skin conditions picked in this study. Acne vulgaris contributed to 7.8% of all skin conditions and was noted in children aged above 12 years reflecting hormonal influence at this age [7]. Only one participant was noted to have psoriasis accounting only 0.96%; this is consistent with reports from other studies [14, 24, 25]. Post-inflammatory hypo and hyper pigmented conditions noted in this study might be attributed to injuries affecting children in this setting including insect bites.

Nutritional status did not influence occurrence of dermatological conditions in this study and similarly no nutritional dermatoses was noted. This is in contrast to expected finding of nutritional dermatoses as a result of limited food supply. Most orphan centres receive donations enabling them to meet some of their needs. Furthermore, our findings of nutritional status of these children may not reflect the exact situation as children ingress into centres differently [15].

Skin examination was carried out by one investigator (senior paediatric resident) who had received training in the dermatology clinic for 8 weeks, this is an important limitation for this study, although conditions were photographed and were discussed with a dermatologist. Socially acceptable responses to questions regarding personal hygienic practices asked in this study are considered as important limitation.

## Conclusions

Skin conditions were prevalent among children in orphanage centres in Dar es Salaam, male children and children aged 6–12 years were more affected. Predominant conditions were infectious accounting 64.9% most of which were fungal infections. Acne vulgaris was the predominant non-infectious condition. Strategies for preventing these conditions in orphanage centres should be put in place. These strategies should involve improved shelter with reduction of overcrowding, strengthening hygienic practices with provision of adequate soaps and timely management of children with skin conditions which can be achieved with regular medical check-up for these children.

## Data Availability

The datasets used and/or analysed during the current study are available from the corresponding author on reasonable request.

## Abbreviations

CPL: Central Pathology Laboratory
MNH: Muhimbili National Hospital
MUHAS: Muhimbili University of Health and Allied Sciences
SSA: Sub-Saharan Africa

## References

[1] Seth D, Cheldize K, Brown D, Freeman EF (2017). Global burden of skin disease: inequities and innovations. Curr Dermatol Rep..

[2] Hay RJ, Johns NE, Williams HC, Bolliger IW, Dellavalle RP (2014). The global burden of skin disease in 2010: an analysis of the prevalence and impact of skin conditions. J Invest Dermatol.

[3] Mahe A (2005) Epidemiology and management of common skin diseases in children in developing countries. WHO, http://whqlibdoc.who.int/hq/ 2005/WHO_FCH_CAH_05.12_eng.pdf.

[4] Bissek AZ, Tabah EN, Kouotou E, Victor Sini V (2012). The spectrum of skin diseases in a rural setting in Cameroon (sub-Saharan Africa). BMC Dermatology.

[5] Yotsu RR, Kouadio K, Vagamon B, N’guessan K, Akpa AJ, Yao A (2018). Skin disease prevalence study in schoolchildren in rural Côte d’Ivoire: Implications for integration of neglected skin diseases (skin NTDs). PLoS Negl Trop Dis.

[6] Ayanlowo O, Puddicombe O, Gold-Olufadi S: Pattern of skin diseases amongst children attending a dermatology clinic in Lagos, Nigeria. Pan African Medical Journal. 2018; 29:162 doi:10.11604/pamj.2018.29.162.14503. 10.11604/pamj.2018.29.162.14503 PMC6057566 30050626

[7] Komba EV, Mgonda YM (2010). The spectrum of dermatological disorders among primary school children in Dar es Salaam. BMC Public Health.

[8] Kiprono S, Muchunu JW, Masenga JE (2015). Skin diseases in pediatric patients attending a tertiary dermatology hospital in Northern Tanzania: a cross-sectional study. BMC Dermatology.

[9] Morantz G, Cole D, Vreeman R, Ayaya S, Ayuku D, Braitstein P: Child abuse and neglect among orphaned children and youth living in extended families in sub-Saharan Africa: what have we learned from qualitative inquiry? Vulnerable Children and Youth Studies 2013, 8:4, 338-352, DOI: 10.1080/17450128.2013.764476. 10.1080/17450128.2013.764476 PMC3929282 24563656

[10] Doni SN, Mitchell AL, Bogale Y, Walker SL (2012). Skin disorders affecting human immunodeficiency virus-infected children living in an orphanage in Ethiopia. Clin Exp Dermatol..

[11] Mokgatle MM, Madiba S (2015). The burden of disease on HIV-infected orphaned and non-orphaned children accessing primary health facilities in a rural district with poor resources in South Africa: a cross-sectional survey of primary caregivers of HIV-infected children aged 5–18 years. Infectious Diseases of Poverty.

[12] Ferie J, Dinkela A, Mbata M, Idindili B, Schmid-Grendelmeier P (2006). Skin disorders among school children in rural Tanzania and an assessment of therapeutic needs. Trop Doct..

[13] Khalifa KA, Al-Hadithi TS, Al-Lami FH, Al-Diwan JK (2010). Prevalence of skin disorders among primary school children in Baghdad Governorate. Iraq. EMHJ..

[14] Sardana K, Mahajan S, Sarkar R (2009). The spectrum of skin disease among Indian children. Pediatr Dermatol.

[15] Yasmeen N, Khan MR (2005). Spectrum of common childhood skin diseases: a single centre experience. J Pak Medical Association..

[16] Karthikeyan K, Thappa DM, Jeevankumar B (2004). Pattern of pediatric dermatoses in a referral center in South India. Indian Pediatr.

[17] Wenk C, Itin PH (2003). Epidemiology of pediatric dermatology and allergology in theregion of Aargau, Switzerland. Pediatr Dermatol.

[18] Tamer E, Ilhan MN, Polat M (2008). Prevalence of skin diseases among pediatric patients in Turkey. J Dermatol.

[19] Nanda A, Al-Hasawi F and Alsaleh QA: A prospective survey of pediatric .dermatology clinic patients in Kuwait: an analysis of 10,000 cases. Pediatr Dermatol1999; 16: 6-11. 10.1046/j.1525-1470.1999.99002.x 10027990

[20] Mostafa FF, Hassan AAH, Soliman MI, Nassar A, Deabes RH (2012). Prevalence of skin diseases among infants and children in Al Sharqia Governorate. Egypt. Egyptian Dermatology Online Journal.

[21] Ameh IG, Okolo RU (2004). Dermatophytosis among school children: domestic animals as predisposing factor in Sokoto, Nigeria. Pakistan Journal of Biological Sciences.

[22] Schmidt A (1997). Malassezia furfur: A fungus belonging to the physiological skin flora and its relevance in the skin disorders. Cutis.

[23] Uneke C, Ngwu B, Egemba O: Tinea capitis and Pityriasis versicolor infections among school children in the southern Eastern Nigeria: the public health implication. The Internet Journal of Dermatology 2005; 4 (2) Number 2.

[24] Abdel-Hafez K, Abdel Art MA, Hofny ER (2003). Prevalence of skin diseases in rural areas of Assiut Governorate, Upper Egypt. Int J Dermatol.

[25] Amer MA and El-Said MM (1990): Common skin diseases in infancy and childhood. Thesis; M.D, Dermatology and Venerology Department, Zagazig University.

